# Fast Room Temperature Very Low Field-Magnetic Resonance Imaging System Compatible with MagnetoEncephaloGraphy Environment

**DOI:** 10.1371/journal.pone.0142701

**Published:** 2015-12-02

**Authors:** Angelo Galante, Raffaele Sinibaldi, Allegra Conti, Cinzia De Luca, Nadia Catallo, Piero Sebastiani, Vittorio Pizzella, Gian Luca Romani, Antonello Sotgiu, Stefania Della Penna

**Affiliations:** 1 MESVA, Department of Life, Health & Environmental Sciences, L'Aquila University, Via Vetoio 10, Coppito, L'Aquila 67100, Italy; 2 Laboratori Nazionali del Gran Sasso, Istituto Nazionale di Fisica Nucleare, S.S. 17 bis km 18910, Assergi, L'Aquila 67010, Italy; 3 Department of Neuroscience, Imaging and Clinical Sciences, G. D'Annunzio" University, Chieti 66100, Italy; 4 ITA S.r.l., Zona Industriale di Pile, SS17, Località Boschetto, L'Aquila 67100, Italy; 5 Institute of Advanced Biomedical Technologies, G. D'Annunzio" University, Chieti 66100, Italy; National Taiwan University, TAIWAN

## Abstract

In recent years, ultra-low field (ULF)-MRI is being given more and more attention, due to the possibility of integrating ULF-MRI and Magnetoencephalography (MEG) in the same device. Despite the signal-to-noise ratio (SNR) reduction, there are several advantages to operating at ULF, including increased tissue contrast, reduced cost and weight of the scanners, the potential to image patients that are not compatible with clinical scanners, and the opportunity to integrate different imaging modalities. The majority of ULF-MRI systems are based, until now, on magnetic field pulsed techniques for increasing SNR, using SQUID based detectors with Larmor frequencies in the kHz range. Although promising results were recently obtained with such systems, it is an open question whether similar SNR and reduced acquisition time can be achieved with simpler devices. In this work a room-temperature, MEG-compatible very-low field (VLF)-MRI device working in the range of several hundred kHz without sample pre-polarization is presented. This preserves many advantages of ULF-MRI, but for equivalent imaging conditions and SNR we achieve reduced imaging time based on preliminary results using phantoms and *ex-vivo* rabbits heads.

## Introduction

Clinical Magnetic Resonance Imaging (MRI) scanners are usually divided into high field (> 0.5 Tesla) and low field ones. The latter usually rely on permanent magnets and operate between 100 mT and 500 mT. At lower values of the magnetic field, scanners can be divided into Very-Low Field (VLF), for field strengths up to 10–20 mT, and Ultra-Low Field (ULF), in the mT-μT range [1, 2]. Both VLF and ULF MRI usually rely on electromagnets to generate the B_0_ field and, if compared with clinical scanners, they share the advantage of reduced hardware complexity, cost and weight to generate both the static background and gradient fields. Due to their limited field strength and to the possibility to realize open configurations, these systems would allow imaging of patient categories not compatible with clinical scanners (pregnant women, patients with claustrophobic syndrome, infants), while providing increased T_1_ intrinsic contrast among different tissues [3]. They are less sensitive to the presence of metals [4], thus opening the technique to patients with metallic implants. Furthermore, VLF and ULF devices allow integration of MRI with other imaging modalities whose hardware is not compatible with high magnetic fields, such as Magnetoencephalography (MEG) [5, 6, 7]. Nevertheless, despite these advantages, they are not commercially available since they do not guarantee, at the moment, the imaging quality to be considered as a valid tool for clinical practice.

The key ingredients in MRI acquisition are spatial resolution, signal-to-noise ratio (SNR) and scan time, which are mutually related. Up to now, ULF-MRI devices were able to acquire images with a resolution in the mm range [6, 8, 9, 10] and reasonable SNR, meeting the criteria for adequate anatomical imaging [11] but with excessive scan times. One of the reasons for such scan duration is intrinsic to the ULF-MRI approach: in fact, in ULF-MRI the polarization induced in the sample is so small that the SNR must be increased through pre-polarization pulses building up the spins magnetization [12]. This approach has demonstrated successful results both at ULF and low field [13, 14]. Indeed, at ULF working frequencies, sample noise does not dominate electronic noise, and the SNR is proportional to the polarization field [15]. The SNR increment due to pre-polarization is counterbalanced by: (*i*) a longer scan time since, in some cases, the pre-polarization pulses can take up to 75% of the total scan time [16]; and (*ii*) the impossibility to adopt sequences based on the longitudinal magnetization recovery during the sequence itself. Another issue related to the pre-polarization technique is the intrinsic 3D nature of the excitation if the pre-polarization and the readout fields are orthogonal to each other and the former is switched off non-adiabatically. With such an experimental approach it is not possible to excite a single slice of the sample and only time-consuming 3D acquisitions can be performed [6, 10, 16]. Even with pulsed techniques, the ULF signal intensity is low and usually ultra-low noise cryogenic detectors (often Superconducting QUantum Interference Devices, namely SQUIDs, working at the liquid helium temperature) are used to detect it. Cryogenic devices increase the complexity and cost of ULF systems, however they seem to be a natural choice in integrated systems such as MEG-MRI devices [6, 10, 16] as the use of SQUIDs is mandatory in MEG. However, ULF MEG-compatible MRI might still be performed using simple copper wire detection coils. Indeed, it was recently shown that room temperature resonant air coil detection can be performed at 2 mT with pre-polarization pulses of 100 mT [17] or even higher [18].

The VLF systems can also be compatible with MEG. After the pioneering years of MRI [19], VLF-MRI has received little attention. If compared with ULF, the higher field strength of VLF increases the signal intensity and permits imaging without the use of pre-polarization pulses, thus shortening the acquisition time. A single VLF image might be recorded in a time comparable with a high field device, albeit with less SNR. If such SNR is adequate to provide useful anatomical images to accompany MEG, VLF-MRI might considerably increase the chances of an effective implementation of integrated MEG-MRI systems.

The above considerations motivate the realization of a VLF-MRI device designed to ensure compatibility with MEG and allow faster imaging than existing ULF-MRI systems. Although the system here described is a small-scale prototype, it is designed considering the technical requirements of a larger system capable of operating on human subjects. The prototype is used to develop non-standard approaches to MEG-MRI, with the ability to detect the MRI signal in a MEG environment with both cryogenic magnetoresistive sensors coupled to a Niobium flux transformer (which was described in [20]) and room temperature resonant coil. The MRI device was designed and realized by the L’Aquila group and ITA srl. It was installed and adapted to the MEG environment by the group at the University of Chieti (UdA) [20, 21] that also performed the MRI acquisitions.

## Methods

### MEG constraints to MRI devices

The main elements of a MEG device (composed of an array of SQUID detectors inside a liquid helium cryostat, installed in a magnetically shielded room) place strong limitations on the design of an integrated MRI scanner [22].

First, the MEG detection channels are based on the extremely sensitive low-T_c_ SQUIDs coupled to magnetometers and/or gradiometers via flux transformers [23]. SQUIDs are non field-tolerant devices that need to be protected from strong fields as well as field transients to prevent flux trapping. This feature limits the measurement field in MEG-MRI devices to a few hundreds of μT [24], when SQUIDs are used as detectors also for the MRI signals. To increase the SNR, these systems adopt additional pulsed fields, up to a few hundreds of mT, and the SQUIDs are protected using suitable strategies [24].

Second, the magnetic field sensors (up to about 300 units for commercially available devices) are hosted in a liquid helium cryostat with a helmet-shaped tail. These cryostats are equipped with super-insulation layers realized with good thermal and electrical conductor. The layers act as a low pass filter for any radiofrequency (RF) signal generated in the sample and prevent RF above some threshold frequency from reaching the cryogenic detectors. This, via the Larmor relation, limits the maximum intensity of the static B_0_ field for any MRI detection system placed inside the cryostat.

Finally the magnetic shielded room (MSR) puts further constraints. The MSR is mandatory as the weak magnetic fields generated by the neural currents are considerably smaller than the fluctuations of the geomagnetic field and the environmental magnetic noise. MSRs are usually composed of several layers of high magnetic permeability material (e.g. μ-metal) and high conductivity material (aluminium) to shield respectively low and high frequency components of the environmental electromagnetic field. The MSR can interfere with a MRI scanner in several ways. If the μ-metal layers are exposed to intense induction fields, they can permanently magnetize (a few Gauss can be enough) depleting their shielding effect. The conductive layers of the MSR can interact with any MRI pulsed field generating shielding currents, creating time-varying magnetic field inside the MSR that can spoil the B_0_ homogeneity or modify the time evolution of nuclear spins [25]. The effect of the eddy currents, just described, is more severe at ULF because of the pre-polarization pulses. Another issue related to the MSR is the filtering of all electrical connections that go through its walls. Usually, in MEG systems installed in a MSR, all the electrical cables through the MSR walls are low-pass filtered to reduce RF inputs, which would impair SQUID operation. Since brain signals comprise frequencies below few kHz, RF filtering is transparent to MEG detection. However, if the same cables are used for the MR signal, the cut-off frequency limits the maximum Larmor frequency (see the next section).

### Design and implementation of the MEG-compatible VLF-MRI system

The choice of a VLF-MRI device is driven by the following reasons: i) to reduce the acquisition time; ii) to avoid pulsed fields to minimally interfere with the MSR; iii) to adopt detectors simpler and cheaper than low T_c_ SQUIDs to detect the MRI signal [26].

In VLF-MRI systems, room temperature resonant coils can be used to detect the MRI signal. Studies on the comparison between same-geometry cryogenic and non-cryogenic surface detection schemes report that room temperature air coils underperform untuned SQUID gradiometers below few MHz and should not be considered the optimal choice at low frequencies [27]. However, if the same-geometry constraint is relaxed, room-temperature resonant volume coils are more versatile as they are placed outside the cryostat and could feature larger filling factors. Another advantage for room temperature resonant coils is the possibility to increase the sensitivity adopting multi-turn configurations to increase the coil inductance. At low frequencies (< 1 MHz), where AC contributions to the coil resistance become smaller, the multi-turn solution is a good strategy. Conversely, in SQUID-based NMR detectors the pick-up coil inductance is limited by the requirement to match the input coil inductance, which is coupled to the micrometric sized SQUID loop. For the above reasons it can be expected that the frequency limit proposed in [27] should shift towards lower frequencies when optimal geometries of the resonant volume coils and SQUID-based detectors are compared.

We present a VLF-MRI proof-of-concept demonstration with a spherical Region of Interest (ROI) of approximately 6 cm. The system is designed to operate inside an MSR and to be scaled to a full-head human device. The MSR (3.0 × 4.0 × 2.7 m^3^) consists of 3 μ-metal layers and one external aluminum layer, for a total attenuation of the external magnetic field of 92 dB at 10 Hz. Additionally, the system is designed to be compatible with a cryostat installed on a non-magnetic gantry placed inside the MSR. The cryostat is a model with a curved bottom of 16 cm radius (manufactured by CTF Systems Inc, Canada). The cryostat super-insulation and thermal shielding determine a transfer function with a low pass filter shape. To allow both cryogenic and room-temperature detection, the measurement field is fixed at 8.9 mT (373 kHz), corresponding to a 10% attenuation of the radiofrequency signal inside the cryostat. As for other existing MEG-MRI ULF realizations [6, 9], the VLF-MRI prototype is designed to allow sequential MEG and MRI acquisitions. Specifically, MRI acquisitions could be performed after the MEG recordings, while MEG could be recorded with the magnet and the RF coils disconnected from the driving electronics to reduce possible noise inputs (see also [6]).

### The field set-up

To generate the static B_0_ field several options are considered: Helmholtz pairs, compensated Helmholtz pairs and an end-compensated solenoid. All setups should be coupled to the cryostat and provide the region of maximum homogeneity close to its bottom, where the ROI is located. For a Helmholtz pair the maximum homogeneity is obtained for a coil radius *r* equal to the coil separation distance *d*. From a power series expansion of the magnetic field along the coils longitudinal axis [28] it can be seen that the 100 ppm homogeneity region (a fair value for VLF-MRI) extends approximately up to a distance of *R* = 0.1 ⋅ *d* from the geometrical center and the total current needed for the target *B*_0_ field in the center is given by ${\left(\frac{5}{4}\right)}^{3/2}B_{0}d/\mu_{0}$. To image a human head with a spherical Field Of Viev (FOV) of radius *R* = 10 cm, a separation of *d =* 100 cm is required for the Helmholtz coils. This coil size is difficult to fit inside a typical MSR. Such large coil structures generate a large stray field and could easily induce permanent magnetization of the close MSR walls. Additionally, assuming copper windings, such coil configuration would require a high driving power: $P∼4\pi \;{\left(\frac{5}{4}\right)}^{3/2}\rho d^{2}B_{0}j/\mu_{0}$ where ρ and *j* are the resistivity and the current density respectively [28]. For reasonable values of *j* (few A/mm^2^) the total amount of power for a human sized system is in the kW range already for B_0_ = 10 mT. An alternative design could adopt compensated Helmholtz pairs to improve the field homogeneity. In this case a second pair of coils has to be added to null the first, second and third derivatives of the B_0z_ field along the z axis in the coils isocenter. Since the compensation coils produce correction fields with opposite directions, the coil system is less efficient in comparison to the simple Helmholtz pair. In this case the total amount of power for the same field strength increases. Tens of kW are needed (for B_0_ = 10 mT) for a human sized set-up and *j* = 3–4 A/mm^2^. Increasing the amount of copper and reducing the current density, it is possible to reduce the dissipated power but a very heavy magnet is needed. In both cases this approach could not fit the majority of existing MSRs.

In conclusion, in order to scale the prototype system to a human-sized MEG-MRI system, ensuring at the same time the compatibility with the MSR, the only viable option is a closed geometry with high efficiency and limited dispersed field. A solenoid fulfills all the requirements. The field homogeneity can be improved by varying the turn density along the magnet axis, to compensate for finite length effects [28] and, if needed, the stray field can be reduced with compensation coils. With this configuration an electromagnet with 60 cm inner bore (to contain the patient head and torso), 120 cm length and 200 kg of weight can produce a 10 mT field with less than 300W of dissipated power, which is still manageable with air-cooling. Thus, the solenoid geometry was adopted for the small-scale prototype, thanks to the possibility to propose a similar system in full-scale set-up.

**Fig 1:**
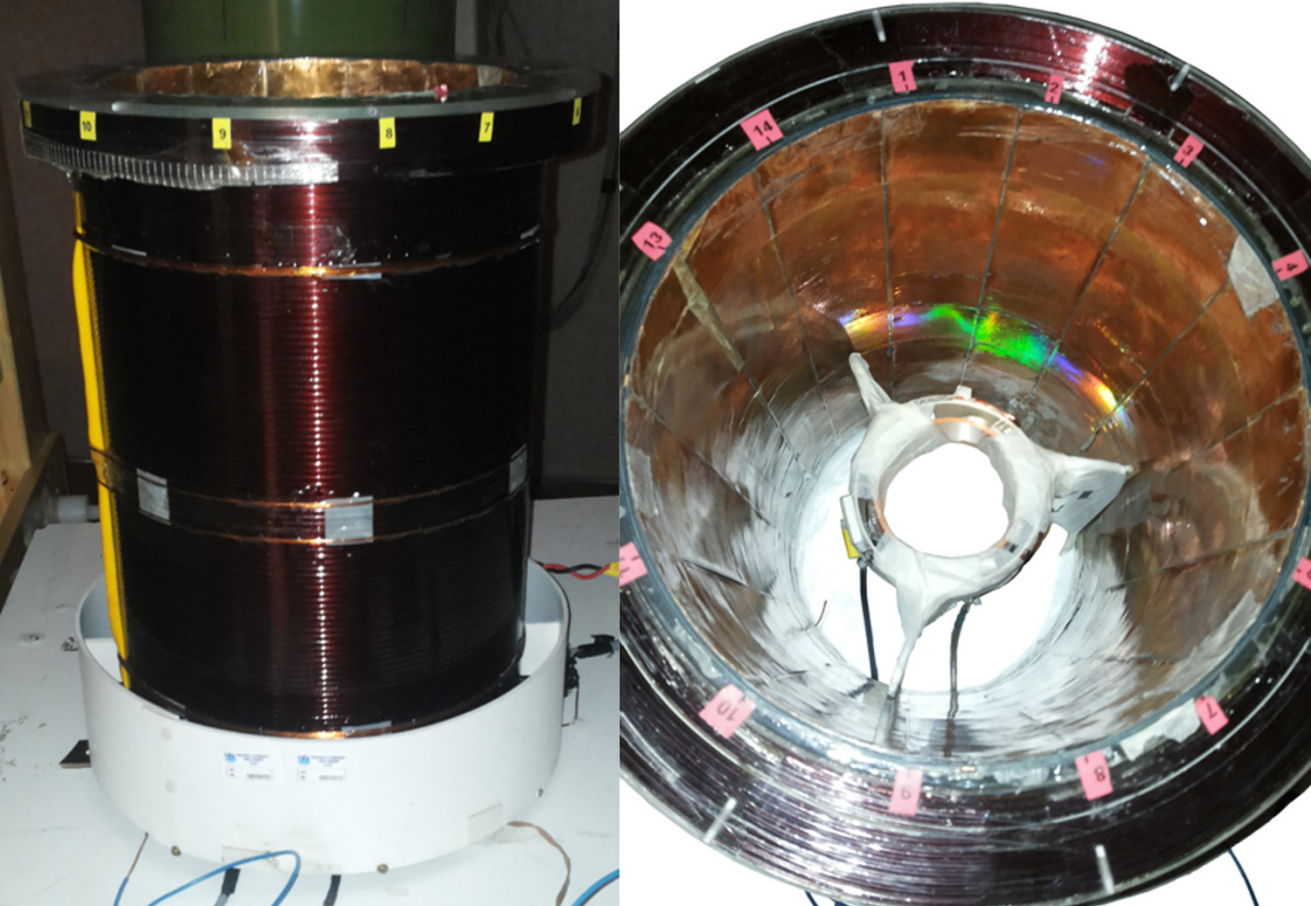
The VLF-MRI coil system. The main compensated solenoid with G_z_ gradient coils (left) and the RF coils placed inside the magnet bore (right).

In the prototype described here, the main coil is a solenoid which contains the cryostat bottom. Thus, the solenoid has a 23.4 cm inner diameter (see Fig 1) with end corrections calculated to null the second and fourth order derivative of the axial field in the center. The coil power supply is a SM 120–13 (Delta Elektronica, The Netherlands), providing a voltage of 16.2 V and a current of 3.85 A to generate 8.9 mT in the solenoid center. An external reference voltage drives the current generator to improve current stability over time. Additionally, the output of the power supply is low pass filtered (outside the MSR) through a home-made high power 5 pole balanced filter with a cut-off frequency of 43 Hz, to reduce fluctuations of the current feeding the solenoid as well as to prevent noise input at the Larmor frequency. The power dissipated to generate 8.9 mT is 62 W, a value that allows continuous operation without any need of cooling with a coil working temperature of 35^°^C for standard room temperature conditions. The measured B_0_ homogeneity inside the ROI is about 150 ppm and, in the operational position, the induction field on the closest MSR wall is, without any compensating coil, below 1 G.

The X-Y gradient coils are designed using a Finite Element method [29, 30] and are located on the inner surface of the solenoid. The resulting continuous current density distribution is discretized using the stream function approach. They produce 0.38 mT/(m A) with 0.2% gradient inhomogeneity in the ROI. The Z gradient is a compensated Maxwell pair configuration placed outside the main coil (see Fig 1), producing 0.22 mT/(m A) with 1% gradient inhomogeneity in the ROI. All the gradient coils are driven by amplifiers from Copley Corp. operated at switching frequency of 71kHz and 6V-22A maximum output, and able to provide a maximum current of 10A in the coils. Thanks to their geometry, the gradient coils generate a field mainly confined within the magnet volume. The maximum field generated by the coils on the closer MSR wall is less than 1 *μ*T, so there is no need for active or passive shielding.

### Transmission and detection coils

Two separate room-temperature resonant coils are used for the transmission (Tx) and for the detection (Rx) of the signal (see Fig 1) to image a spherical volume with 6 cm diameter. The Rx coil is a 27 turn saddle coil made of Litz wire wound on a plastic cylinder with 8 cm diameter and a Q of 105. The Tx coil is another saddle coil made with 3 turns of standard copper wire and a small Q value to reduce dead time after RF pulses. The two coils are 90^°^ rotated so to achieve a 60 dB decoupling. A single ended RF power amplifier drives the Tx coil (ITA, http://www.imagtech.it/), it is placed outside the MSR, with 42 dB gain and 25 W maximum power into a 50 Ohm load. The Rx coil is connected to a battery supplied preamplifier located inside the MSR to avoid ground loops with the MSR and to reduce the noise that might be introduced by the supply line. The preamplifier is a single ended, custom design with 60 dB gain and 2 dB noise figure from ITA srl.

The MRI signal is received by a ITA srl console (up to 4 receive channels, 14 bit and 40 MHz sampling frequency, one transmit channel), connected to a host PC via Ethernet.

### Strategies to make the MRI set-up MSR-compatible

The console, the power supply for the main solenoid, the gradient and RF amplifiers are placed outside the MSR. They are connected to the related hardware components inside the MSR by filtered throughputs, with a cutoff frequency of about 1.5 MHz. Additionally, to reduce the noise at the measurement frequency (which may be carried by the cables or might be due to ground loops between supplies and the MSR throughputs) the following strategies are adopted:

- All cables are shielded outside and inside the MSR, to reduce possible inductive effects between the rest of the instrumentation and the RF signal pick-up and transmission line.
- The console ground is floating and the Ethernet connection to the driving computer is de-coupled through a passive network isolator (Phoenix Contact, Germany, FL ISOLATOR 100-RJ/RJ).
- The power line driving the current generator for the generation of the static B_0_ field is decoupled from the laboratory ground through a 1:1 transformer, to avoid ground loops.
- The power supply of the digital interface of the gradient amplifiers is decoupled from the laboratory ground, to avoid loops with the power supply of the gradient amplifiers. Linear power supplies instead of switching supplies are used for the digital interface.
- Low pass filters with cut-off frequency of 20 kHz are applied to the three gradient cable pairs to avoid noise input.
- The output of the Rx preamplifier and the console receive channel are decoupled from the MSR filters through a transformer (type UT10211LS).
- The RF signal generated by the console is coupled to the RF amplifier through a transformer, and the amplifier output is coupled to the Tx coil through a second transformer to avoid ground loop with MSR filters.
- Since the RF amplifier output is noisy also when the amplifier is not transmitting, a pair of blocking diodes are installed before the transformer, to avoid SNR degradation of the NMR signal. Further, mechanical switches (COTO Technology 9012-05-10) are installed in series with the RF amplifier to isolate it from the receiving channel. Their switching time is about 0.35 ms. The MRI console triggers the switches through an optically decoupled TTL signal (optical decoupling circuit is also battery supplied). Specifically, in a spin-echo sequence, they are opened at the end of the refocusing RF pulse.

### Tests on compatibility with a MEG channel

**Fig 2:**
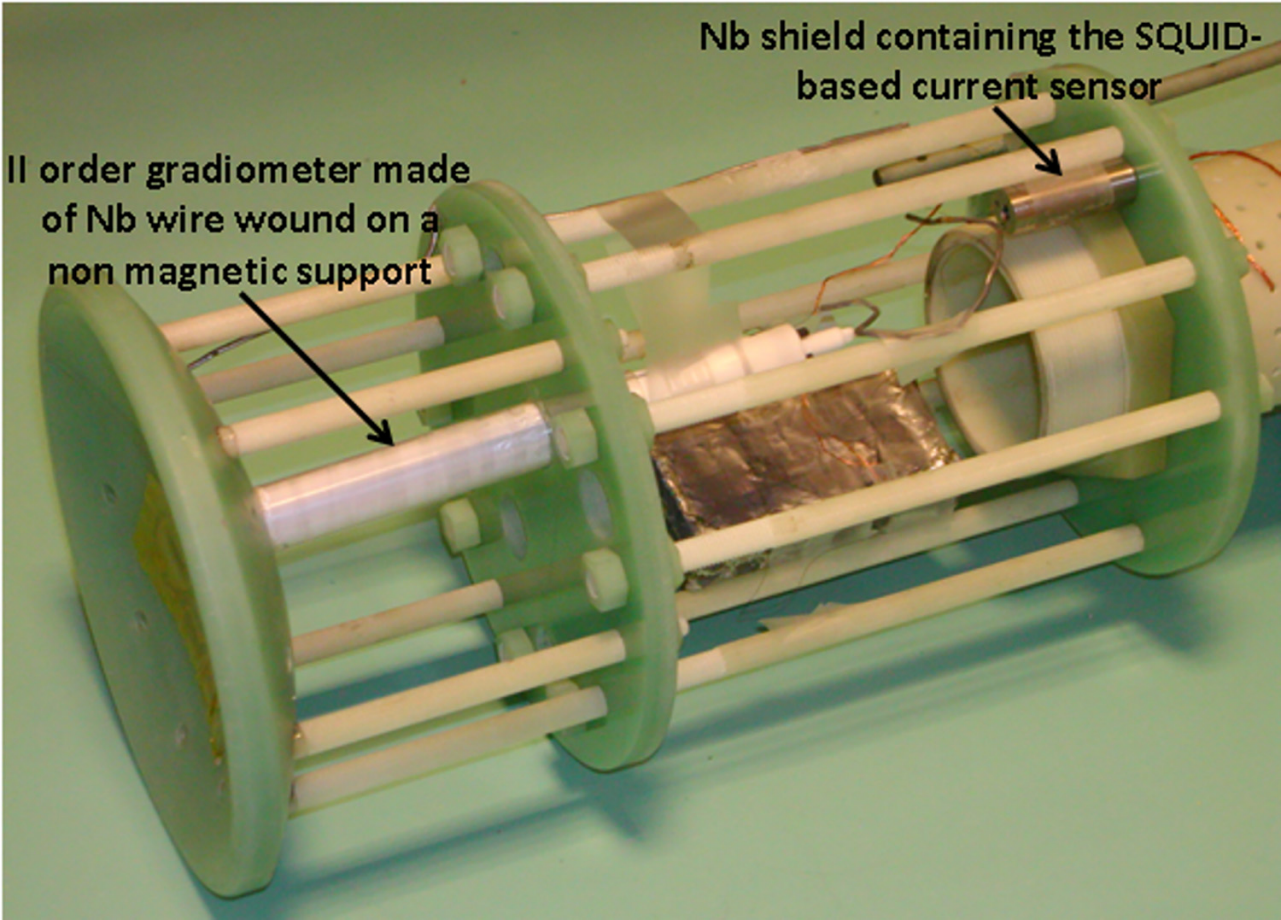
MEG channel consisting of a superconducting II order gradiometer coupled to a dc SQUID. Both the superconducting connections and the SQUID are placed inside superconducting shields. The MEG channel is mounted on the same probe supporting the superconducting detector described in [20].

To provide evidence that the MEG data quality is not affected by the presence of the MRI equipment (when it is suitably powered off), signals from a MEG channel with and without the MRI set-up in the measurement position were recorded and compared. Notably, as the size of the RF coil size prevents the possibility of recording human MEG signals, only the noise in the MEG channel was measured. The MEG channel consists of a Niobium wire-wound second order gradiometer with a diameter of ∼1.5 cm and a baseline of ∼8 cm. The gradiometer is connected to a current sensor consisting of a dc SQUID coupled to a superconducting input coil integrated on the same chip (model CS2Blue, Supracon AG, Germany). The gradiometer is mechanically connected to the input coil through superconducting washers and screws. The superconducting contacts and the current sensor are installed inside Niobium shields. The MEG channel is mounted on the same probe used to test the NMR superconducting R_X_ channel reported in [20], as shown in Fig 2. The probe is inserted in the cryostat tail. The dc SQUID is driven by a direct readout scheme. The MEG channel output is band-pass filtered at 0.16–270 Hz and sampled at 1024 Hz. During MEG noise recordings, the MRI set-up is unplugged from the connectors to the driving electronics. The rationale for not connecting the field generators/receivers to the driving electronics is to prevent input of magnetic noise generated by wires connected to instrumentation in the OFF condition, during which the overall circuitry might be different from the optimized ON condition. The Power Spectrum Density (PSD) is estimated using a Hanning window of 16384 samples, with a resulting frequency bin of 0.0625 Hz.

### MRI phantoms and sequences

Two phantoms are used to test for geometrical distortions and for the acquisition of reference images. The former is a plastic regular grid (8 mm high, square holes of 6 mm), held by a cylindrical container (50 mm diameter, 12 mm height). The latter has an asymmetric 3D structure, 42 cm^3^ volume and total size of 5x5x4 cm^3^. Both phantoms are filled with doped water (1000 ml H_2_0, 770 mg CuSO_4_, 1 ml arquad, 0.15 ml H_2_SO_4_) with T_1_ = 130 ms at the working frequency.

Robust spin echo sequences are used for both 2D and 3D acquisitions, recording a single echo for each spin excitation. All images are obtained with hard RF pulses lasting 200 μs. The pulse BW is in the kHz range and guarantees a uniform sample excitation inside the ROI. Short echo times (T_E_ = 19 ms) are used to maximize the SNR with T_R_ = 500 ms or T_R_ = 300 ms. The 0.35 ms delay introduced by the switching time of the relays is negligible for the selected T_E_, but its effect should be investigated if much shorter echo times are needed. In all the images, the noise level is computed using the variance of the background voxels.

Fast 2D acquisitions without slice selection (a projection of the phantom on a 2D plane) are used to compare a polar raster sampling of the K-space (reconstruction from projections) and standard Cartesian sampling. Images are recorded with 32x32 voxels, 3 mm voxel size, T_R_ = 500 ms and a single acquisition (NEX = 1). K-space is sampled with a full Cartesian (32 encoding steps, T_acq_ = 16 s) and an under-sampled polar (16 projections, T_acq_ = 8 s) raster. 2D phase encoded acquisitions are also tested for geometrical distortions with 1x1 mm^2^ voxel size (64x64 voxels) and Cartesian sampling.

For 3D images only Cartesian sampling with two phase-encoding gradients is used. This is the natural strategy to push the spatial resolution to 1 mm avoiding the gradient-based limitations of too long slice selection RF pulses. Single scans of 32x32x32 voxels with isotropic 3x3x3 mm^3^ resolution, 9.6 cm FOV and T_R_ = 500 ms are acquired in 8.5 min. A 32^3^ image with a nominal resolution of 2x2x2 mm^3^ and 6.4 cm FOV could be acquired in 8.5 min (no zero-filling). However, improving the resolution beyond 3 mm would require to increase the NEX in order to avoid a dramatic SNR drop. The system can provide a 64^3^ image matrix with 1x1x1 mm^3^ resolution and 6.4 cm FOV, but the scan time would become very long (each full K-space acquisition would take 34 minutes) and the B_0_ drift effects (determined by the limited stability of the current source) would become clearly evident even for a single scan. For the above reasons the acquisition time of the highest resolution images are reduced to 8.5 min using only 32x32 phase encoding steps and zero filling. The latter represents the only processing performed on the raw data motivated by the need for a balance between recording time (total number of phase encoding steps) and image quality. The 3 mm and 1 mm isotropic resolution images are obtained with 260 Hz/cm and 780 Hz/cm reading gradient respectively.

Ex-vivo images of two rabbit heads were acquired few hours after the rabbit sacrifice and the separation of the head from the body. The sample did not undergo any treatment or tissue fixation. The VLF field images of the first head were acquired with the isotropic 3 mm resolution sequence discussed above, T_R_ = 500 ms and NEX = 16 for a total acquisition time of 2.3 h. The high field images of the rabbit were obtained with a 3T scanner (Achieva, Philips) using a knee coil and 3D T_1_-TFE (Ultrafast Gradient Echo) standard clinical sequence for brain anatomical characterization with 1x1x1 mm^3^ resolution, 12x12x18 cm^3^ FOV, T_R_ = 8.5 ms, T_E_ = 3.9 ms, NEX = 3 and T_acq_ = 6 min. The high field image is down sampled in the image space to match the low field resolution and spatially co-registered using an in-house algorithm [31]. The second rabbit head was scanned with the same spatial resolution as the first one (both at VLF as well as at HF), but with T_R_ = 300 ms and NEX = 9, for a total acquisition time of 46 min.

## Results

We report the performance of a VLF-MRI scanner using only room-temperature Tx and Rx resonant coils. The first results obtained using a superconducting NMR coil coupled to a mixed sensor have been reported elsewhere [20].

**Fig 3:**
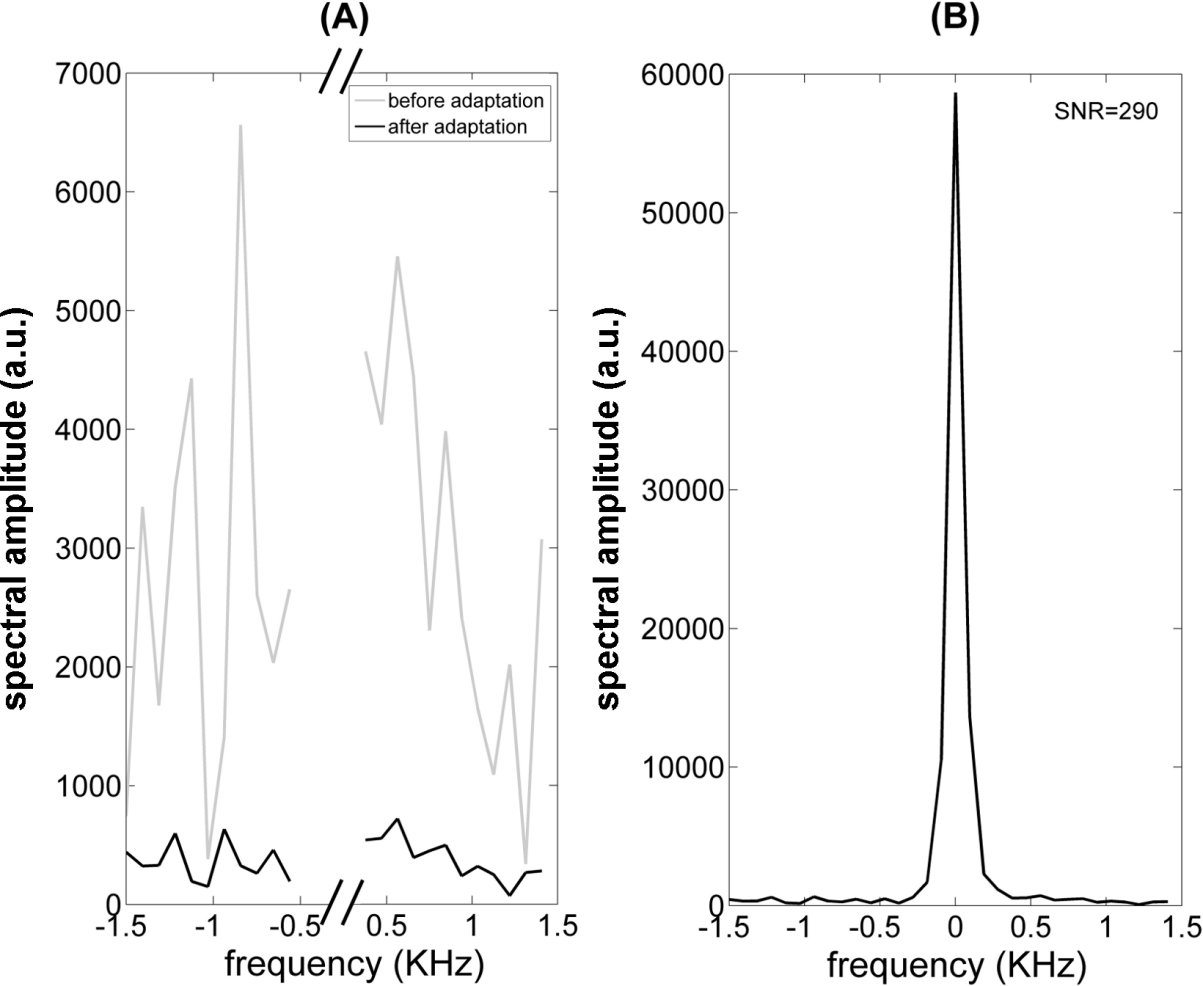
Spin echo recordings (T_E_ = 19 ms, T_R_ = 500, NEX = 10): (A) system noise before and after the improvements done in the integration of the MRI system with the MSR; (B) an echo recorded in the final configuration. The 10-fold noise reduction provides strong evidence of the care that has to be used in integrating a VLF-MRI device with a MSR designed for MEG measurements.

**Fig 4:**
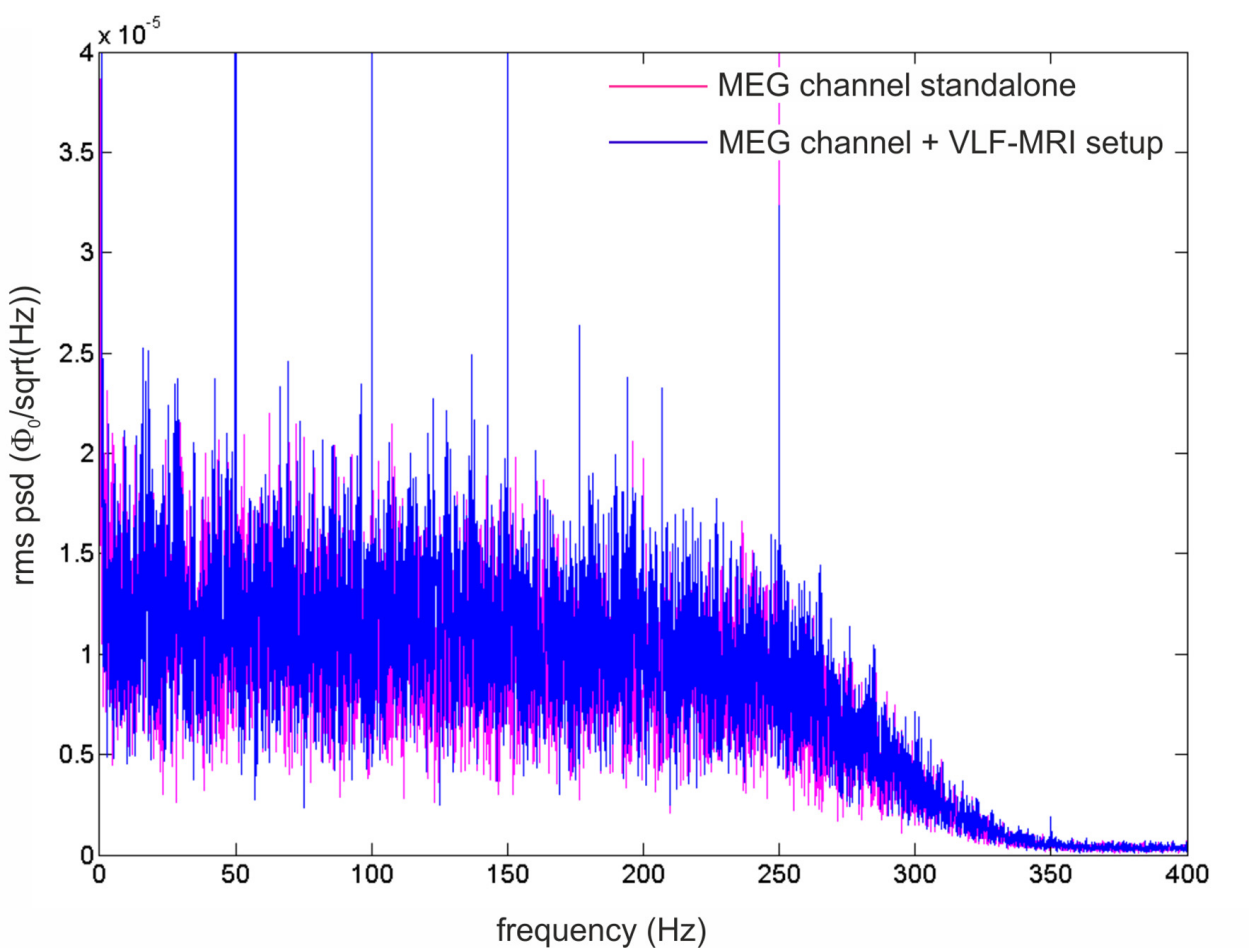
Rms PSD (root mean square power spectrum density) of a MEG channel (band-pass filtered at 0.16–270 Hz and sampled at 1024 Hz, ϕ_0_ is flux quantum = 2.07^−15^ Wb) obtained when the MRI set-up (magnet and RF coils) is placed in the measurement position (blue rms PSD) and when it is placed at about 2 m from the MEG channel (pink rms PSD). The effect of the MRI setup is limited to a 3% increase of the mean white noise. The 50 Hz peaks (and harmonics) are present in both conditions and are not modulated by the MRI setup in the measurement position.

First, compatibility with the MSR environment was tested. Specifically, to evaluate the efficacy of the grounding and filtering strategies discussed in the previous section, the noise standard deviation was measured before and after their implementation. Using the same scan parameters, the noise standard deviation in single echo acquisitions (10 averages) was reduced from 1638 to 167 (two-tail t-test, p < 2e^-7^, see Fig 3A). The 10-fold noise reduction provides a strong evidence of the care that has to be used in integrating a MRI device with a MSR designed for MEG measurements. A sample echo was obtained using the same settings as for the noise estimation (see Fig 3B). We tested that the noise is not affected by the presence of the sample since, as expected for such low fields, it is dominated by the receive channel. Then, a quantification of the effects the MRI hardware induces on a MEG channel was obtained as in the following. The noise of the MEG channel was recorded (*i*) in the MSR when the MRI set-up (magnet, gradients and RF coils) was placed in the measurement position (magnet wrapping the cryostat tail and top of the RF Rx/Tx coils touching the tail bottom) and (*ii*) soon after the MRI set-up was placed at about 2 m from the MEG channel. This test allows us to quantify the unavoidable contribution of close-by conducting elements. The PSDs are shown in Fig 4. The average of the PSD from 51 Hz to 99 Hz was used to evaluate the effect of the MRI setup. Within this band, a 3% increase in white noise is obtained; such a small change in the noise level is not expected to impact MEG data quality. Notably, peaks at 50 Hz (and harmonics) in the PSD (due to the residual environmental field in the MSR), were not significantly modulated by the MRI setup (data not shown).

**Fig 5:**
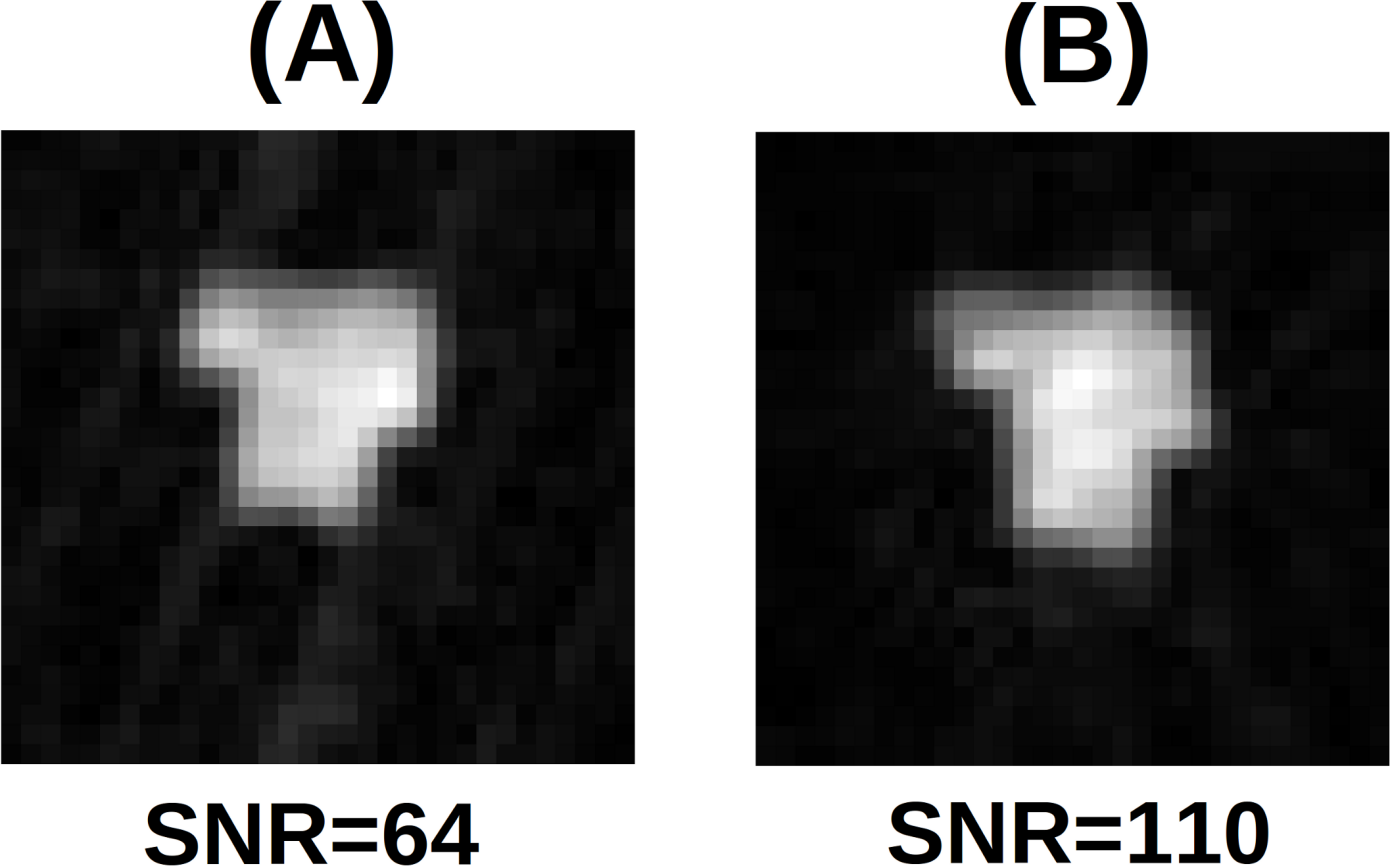
2D phantom projection (spin echo, T_E_ = 19 ms, T_R_ = 500 ms, NEX = 1, no slice selection) with 3x3 mm^2^ resolution: (A) Cartesian (T_acq_ = 16 s) and (B) polar sampling (T_acq_ = 8 s). Cartesian sampling provides lower SNR but less blurring artifacts.

**Fig 6:**
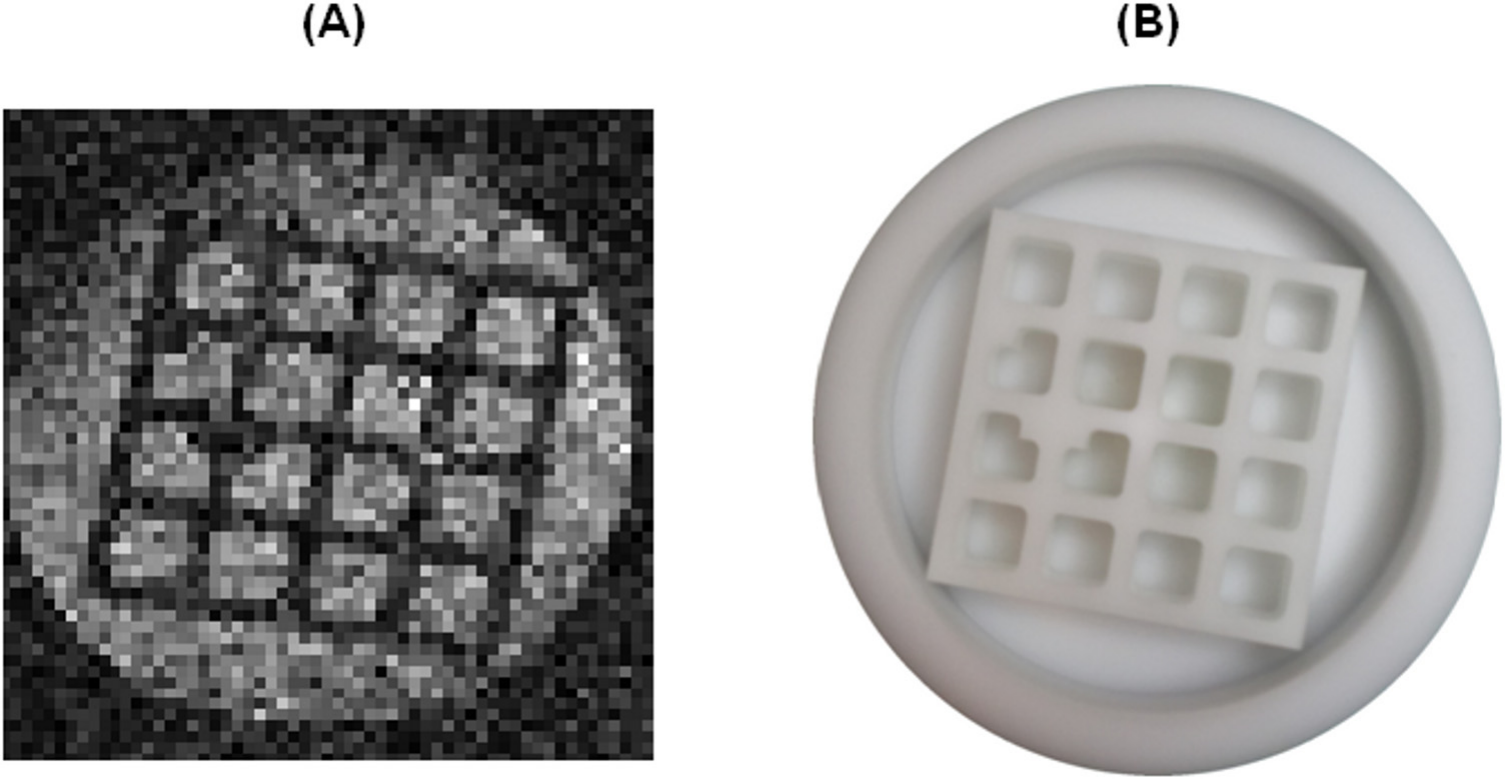
2D phantom projection with 1x1mm^2^ resolution (spin echo, T_E_ = 19 ms, T_R_ = 500 ms, NEX = 500, no slice selection, T_acq_ = 2.2 h) of the linearity phantom (a) and a picture of it (b). There is no evidence of spatial distortions due to concomitant gradient effects.

Second, the suitable strategy to obtain images was assessed. In Fig 5 the 2D projections with 3 mm voxel size and NEX = 1 are visible. Even with half the acquisition time, the SNR of the (undersampled) polar acquisition is 110, considerably larger than the Cartesian data SNR of 64. However, severe blurring impacts the edges of the phantom in the polar image making the sample contours poorly defined (Fig 5B). The effect is only marginally due to the k-space under sampling of the polar acquisitions. Additionally polar reconstruction is sensible to B_0_ instability and imperfections of gradient fields, which produce blurring in the image. For this reason, although the reconstruction from projection can have better SNR and reduced acquisition time [32], only the standard Cartesian sampling with the use of phase encoding gradients is adopted in the following measurements. After selecting the K-space sampling strategy, the 2D phase encoded acquisitions were tested for geometrical distortions with 1x1 mm^2^ voxel size (see Fig 6). In contrast to ULF MEG-MRI [16, 33], significant image distortions are not observed and corrections for concomitant gradients are not necessary.

**Fig 7:**
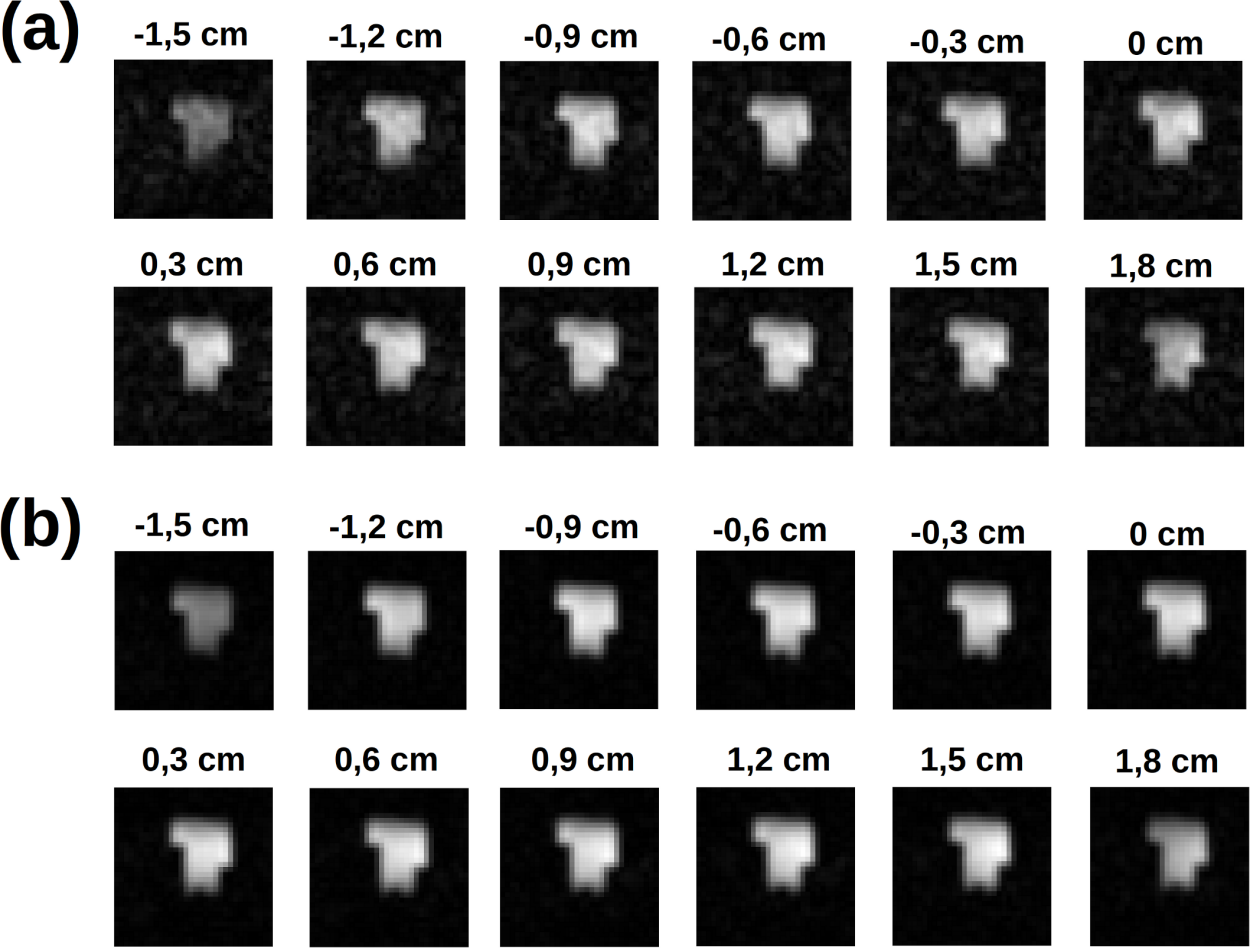
Slices from a 32^3^ 3D phantom acquisition at 3x3x3 mm^3^ spatial resolution (spin echo, T_E_ = 19 ms, T_R_ = 500 ms, 32x32 phase encoding steps): (a) NEX = 1 for T_acq_ = 8.5 min and (b) NEX = 9 for T_acq_ = 77 min. The geometry of the phantom can be clearly detected through 3D VLF-MRI.

**Fig 8:**
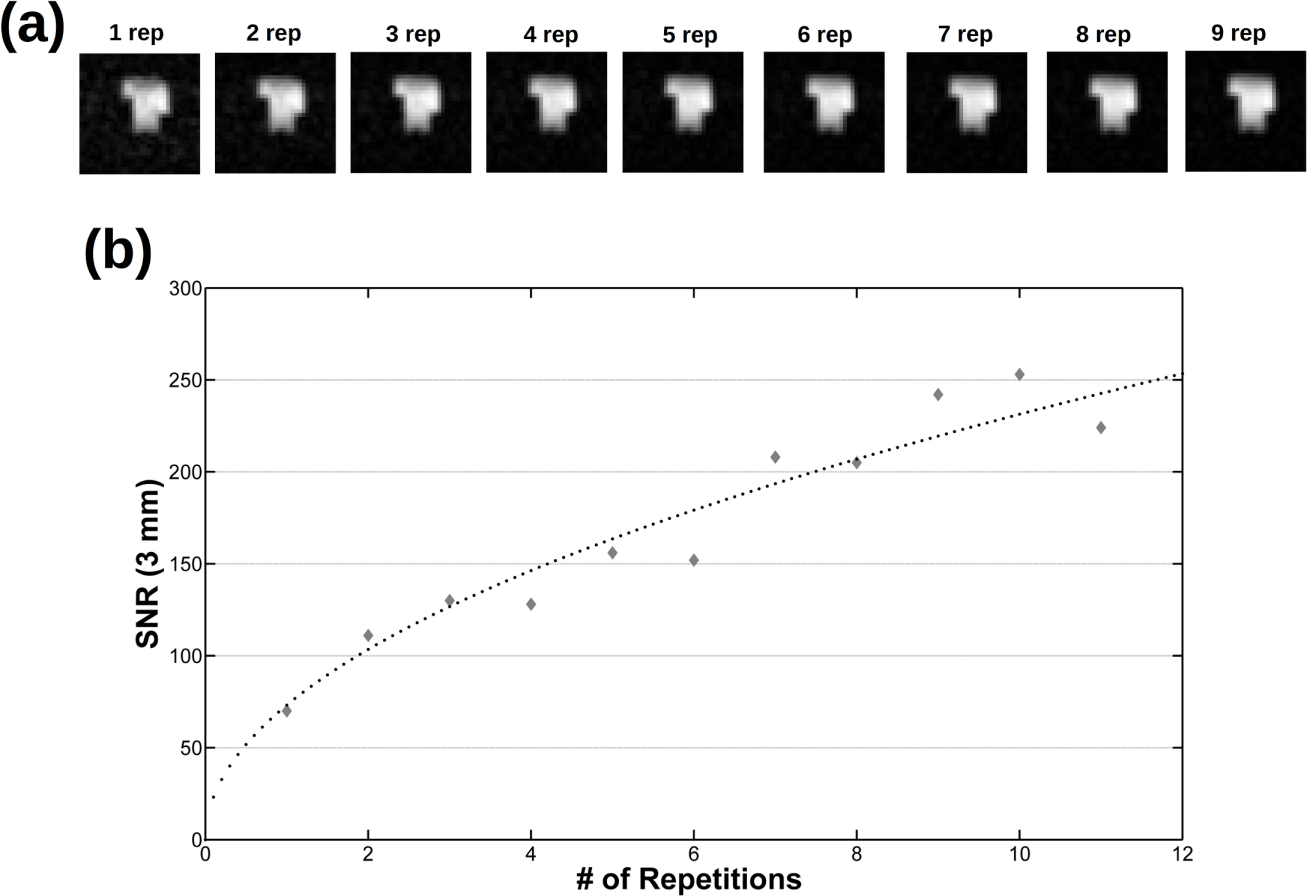
Behaviour of the SNR as a function of NEX. Same acquisition as in Fig 7: the same slice at different NEX values (a) and the corresponding measured SNR (b), together with the related $const\;*\;\sqrt{NEX}$ fit (dotted line).

**Fig 9:**
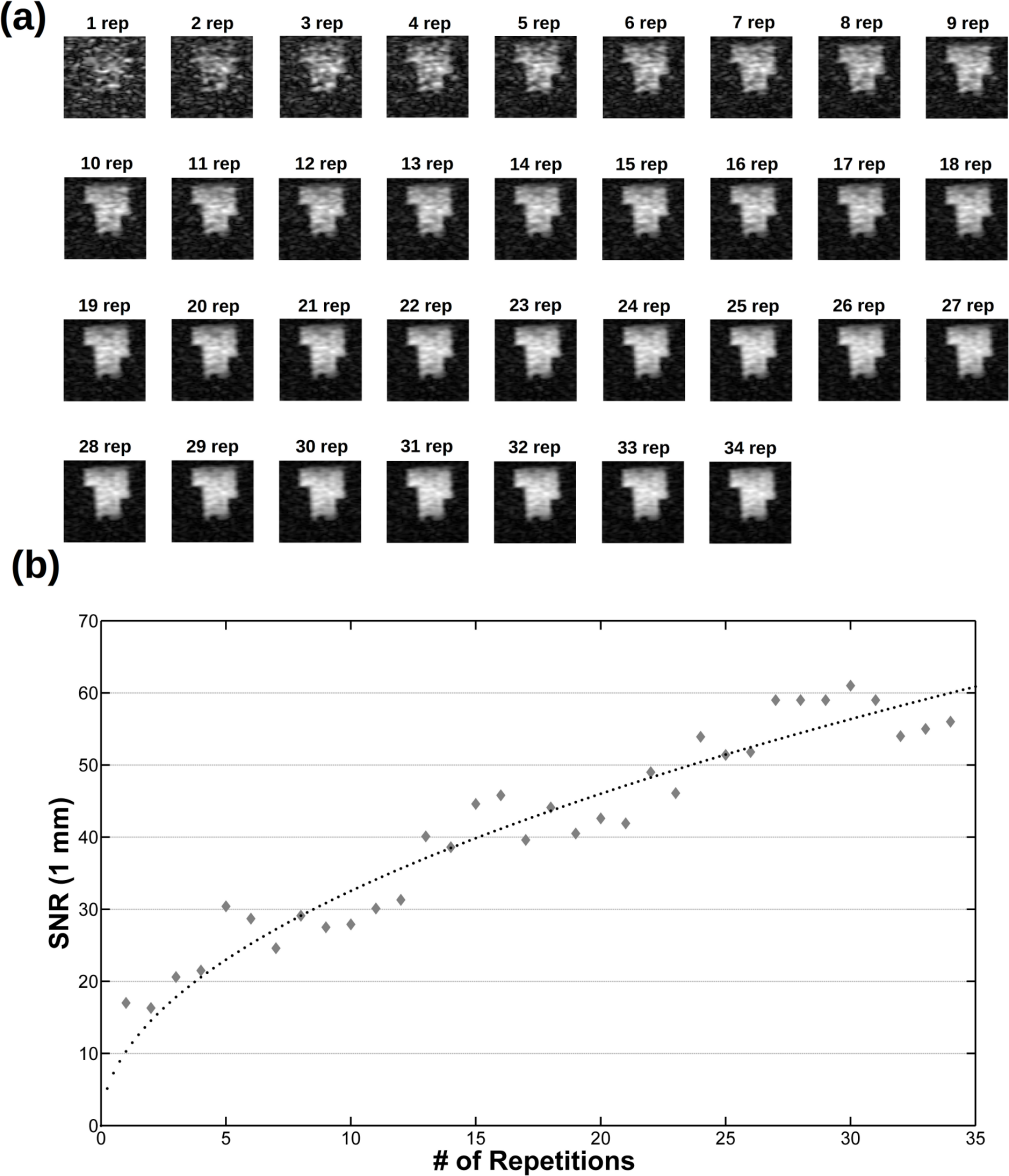
The same slice from a 643 3D phantom acquisition at 1x1x1 mm3 spatial resolution (spin echo, TE = 19 ms, TR = 500 ms, 32x32 phase encoding steps with zero filling to get a 643 matrix data, Tacq = 8.5 min for NEX = 1) at (a) different NEX values and (b) the corresponding SNR together with the related $const\;*\;\sqrt{NEX}$ fit (dotted line). Although a longer acquisition time is needed, images with a resolution of 1 mm^3^ can be recorded with the VLF-MRI system.

Then, images of phantoms and ex-vivo samples are used to assess the imaging performances of the instrument. Fig 7 shows several phantom slices at different depths of the 3D phantom and isotropic 3 mm resolution. The fading effect visible in the upper part of the image is due to the sensitivity profile of the RF coils along the vertical direction (the magnetic field axes) and it can be avoided by increasing the height of the saddle coils. The image analysis shows that SNR = 70 for NEX = 1 and SNR = 224 for NEX = 11 (Fig 8). In Fig 9 a single slice from a 1 mm isotropic 3D acquisition is reported at various NEX values together with the measured SNR. Higher spatial resolution would be possible with currents larger than the 10 A allowed by the gradient amplifiers (or reducing the Hz/cm value but with increasing geometric distortions) but the low SNR is the factor which effectively limits the spatial resolution.

**Fig 10:**
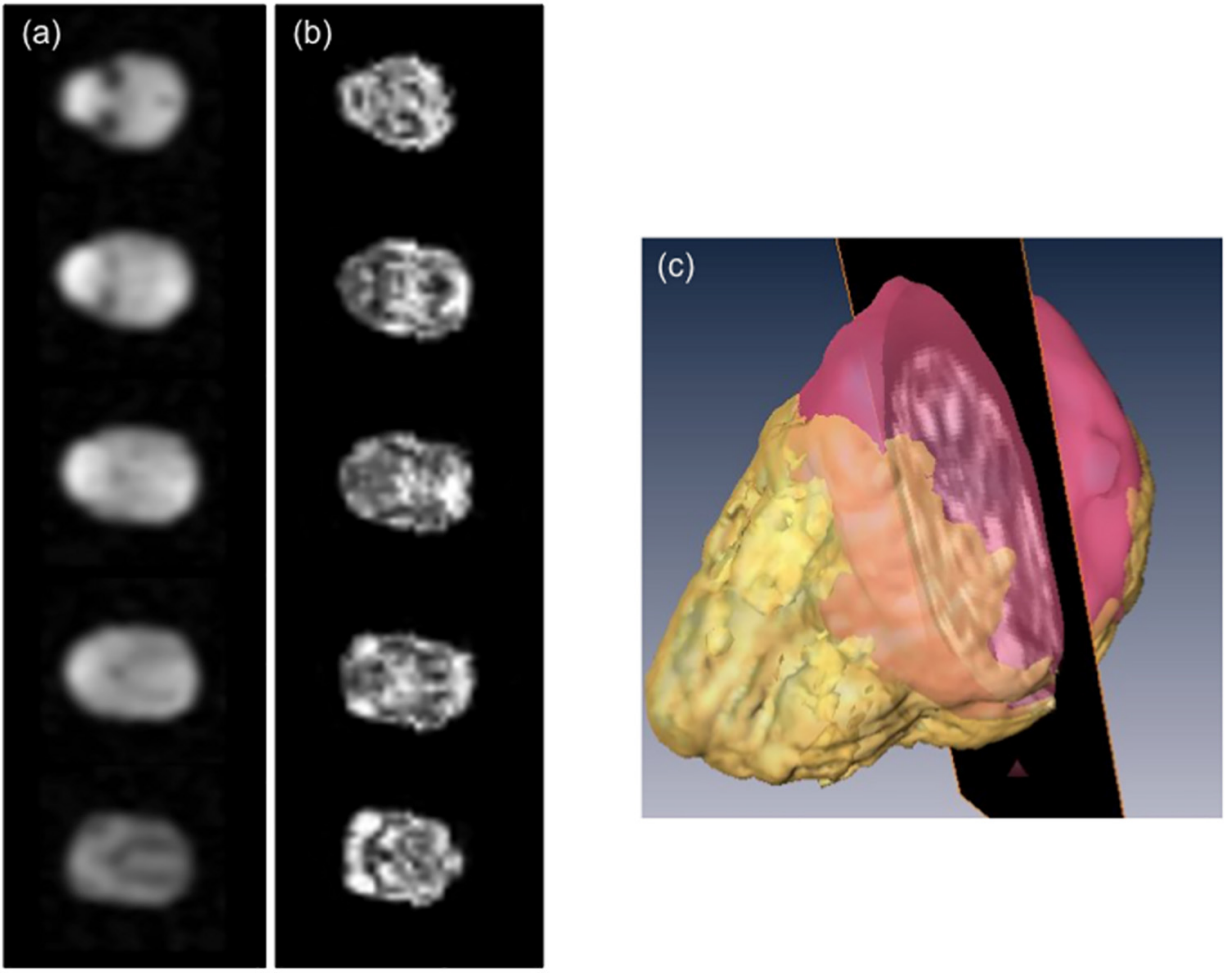
Slices from *ex-vivo* rabbit brain acquisition at 3x3x3 mm^3^ spatial resolution. The slices compare (a) VLF-MRI (spin echo, T_R_ = 500 ms, 32x32 phase encoding gradients, NEX = 16, T_acq_ = 2.3 h) and (b) HF-MRI 3D T_1_-TFE (Ultrafast Gradient Echo) standard clinical sequence for brain anatomical characterization with 1x1x1 mm^3^ resolution, 12x12x18 cm^3^ FOV, T_R_ = 8.5 ms, T_E_ = 3.9 ms, NEX = 3 and T_acq_ = 367 sec. The high field images are down sampled to match the low field resolution and spatially co-recorded. (c) The full 3D co-recorded volumes are shown with pink (VLF) and gold (HF) colors. Despite the lower resolution, VLF-MRI can be co-registered to the related HF-MRI.

**Fig 11:**
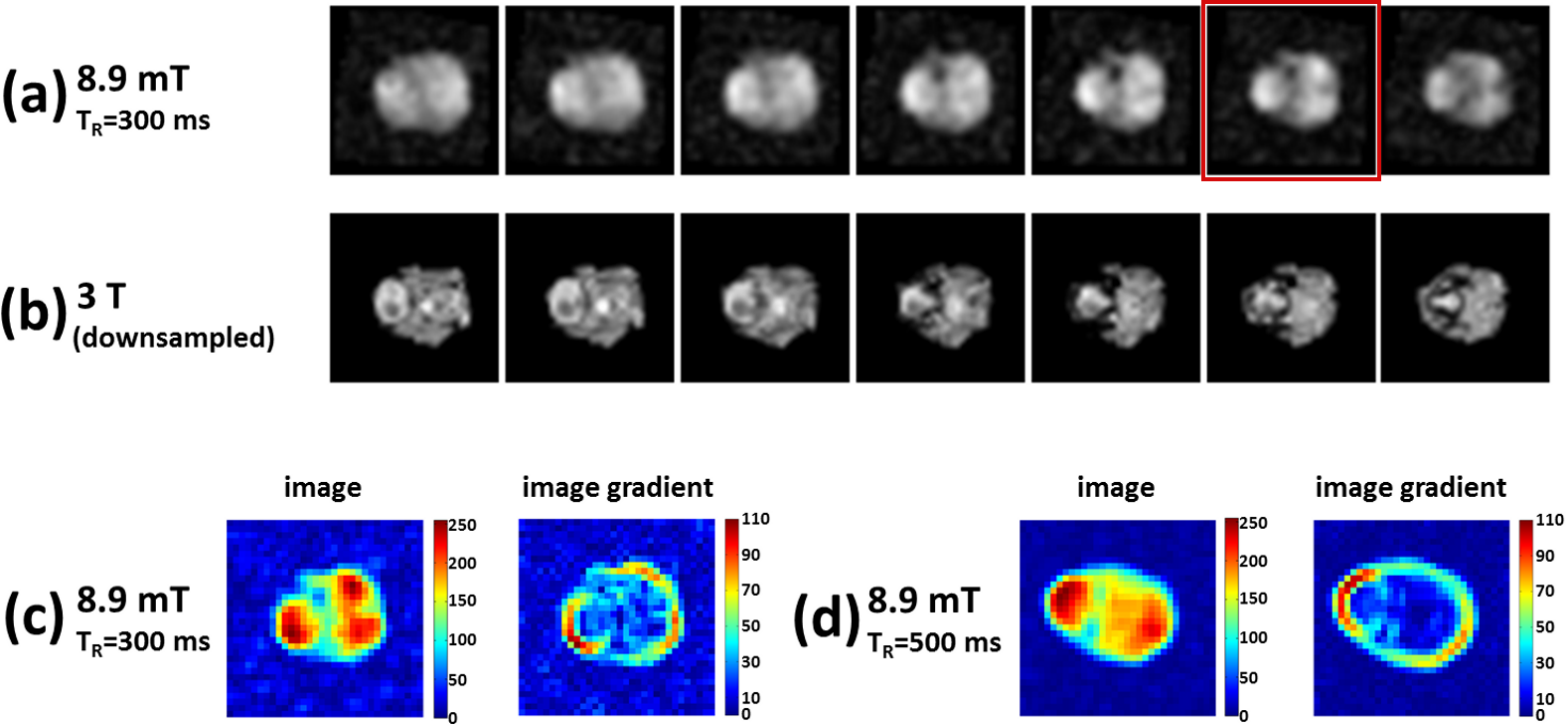
Slices from a second *ex-vivo* rabbit brain acquisition at 3x3x3 mm^3^ spatial resolution. The slices compare (a) VLF-MRI (spin echo, T_R_ = 300 ms, 32x32 phase encoding gradients, NEX = 9, T_acq_ = 46 min) and (b) HF-MRI 3D T_1_-TFE as in Fig 10. Two homologous VLF slices of the two rabbit heads, the second slice from the top in Fig 10A (T_R_ = 500 ms) and the sixth slice from the left in Fig 11A (T_R_ = 300 ms, with a red frame), are selected. The selected slices and the related gradient images are shown in (c) and (d). The image with shorter T_R_ highlights a larger number of edges inside the rabbit brain than the one with longer T_R_ i.e. the increased tissue contrast helps in delineating structures inside the rabbit head.

Finally, a comparison of VLF and HF MRI images using a rabbit head was performed. With T_R_ = 500 ms and NEX = 16 the VLF total acquisition time is 2.3 h and the image SNR is 149. In Fig 10 the VLF-HF MRI comparison of several slices as well as the full co-registered 3D structures are shown. The satisfying overlap of the two image series suggests that the information content of the VLF image can be suitably interpreted by the processing software co-registrating the two image sets. A second rabbit head was scanned with the same spatial resolution as the first one, but with T_R_ = 300 ms, NEX = 9, for a total acquisition time of 46 min. A set of VLF slices, together with the corresponding HF ones, is shown in Fig 11A and 11B. The scan was also used to test, on a qualitative basis, the increase of T_1_ contrast between VLF-MRI acquisitions with different T_R_ values. Two homologous VLF slices of the two rabbit heads, the second slice from the top in Fig 10A (T_R_ = 500 ms) and the sixth slice from the left in Fig 11A (T_R_ = 300 ms), were selected. The gradient images of both slices are shown in Fig 11C and 11D. The image with shorter T_R_ highlights a larger number of edges inside the rabbit brain than the one with longer T_R_. Although the SNR in Fig 11A is smaller than in Fig 10A, the increased tissue contrast of the smaller T_R_ helps in delineating structures inside the rabbit head.

## Discussion

This work shows that a low field MRI device without pre-polarization can be designed to be compatible with the MEG environment, to fit an existing MSR and to produce images at low resolution in a reasonable time. The key ingredient is a coil with closed geometry efficiently generating the B_0_ field while producing low stray fields on the MSR walls. Since MEG users are accustomed to open and full access devices, closed geometry configurations could be considered a disadvantage. However, a suitable design of the set-up allows locating the MEG cryostat inside the solenoid, thus reproducing the standard configuration of a HF-MRI scanner with the RF coils designed to remain as close as possible to the patient head. Indeed, fMRI researchers have developed several ways to provide different stimuli to the subject and similar techniques could be adopted for VLF MEG-MRI with closed coil geometry in a MSR.

To elucidate the advantages of a VLF–MRI system, the SNR results as well as the scan time are compared with: (i) available data from a ULF-MRI system with pre-polarization and room-temperature detection coils [17]; (ii) a ULF-MRI system using multiple superconducting untuned detection channels [10]. Since each system has different geometry and acquisition parameters, suitable normalization factors have to be used to compare them.

The theoretical SNR dependence on the imaging parameters can be written as:

$$SNR\propto \Delta x\Delta y\Delta z\sqrt{\frac{N_{PE1}\;N_{PE2}\;N_{FE}}{BW}NEX}$$ (1)

where Δx, Δy, Δz are the voxel size, N_PE1,2_ the phase encoding steps, N_FE_ the frequency encoding steps and BW the received bandwidth. The expression (1) correctly relates the experimental SNR values of 1 and 3 mm acquisitions of Figs 8 and 9. In the following it will be used to scale the SNR measured with VLF-MRI to the one expected for the acquisition parameters of the two reference systems.

To obtain the normalization factor accounting for the different Rx coil geometries, the reciprocity principle is used. It states that the MRI signal is proportional to the B_1_ value generated by the receiving coil for a unit current. For a Rx coil in saddle configuration, this value is approximately proportional to the inverse of the winding cylinder radius *r*, such that the signal amplitude varies as 1/*r*.

At very low frequencies, the sample noise is negligible and the Rx coil resistance dominates. Besides the effect of resistivity of the coil wire (the DC contribution), the increase of the current density both with the distance from the wire axis (skin depth effects) and with the distance from nearby windings due to the Lorentz force (proximity effects) contribute to increase the resistance as the frequency increases [34]. For a given coil geometry, the DC, skin-depth and proximity contributions are proportional to the coil’s wire length. Up to the tens of kHz regime the DC coil resistance dominates. This dominance can be extended by the use of Litz wire to minimize the skin effect and by optimal choice of the winding spacing [34]. In summary, the Rx saddle coil resistance is proportional to its wire length and hence to the coil scale factor *r*, thus the noise amplitude ∝ *r* ^1/2^. Considering both the Signal and Noise behavior, the expected *r* dependence of the Signal/Noise can be written as:

$$SNR\propto r^{-3/2}.$$ (2)

### Comparison with the ULF-MRI system in [17]

First, the performance of the 8.9 mT VLF-MRI is compared with data from the system described in [17], which uses room temperature resonant coil detection at *B*_0_ = 2 mT, pre-polarization with *B*_*p*_ = 100 mT pulses and 13x13x6 cm^3^ FOV. In [17] the authors reported the SNR values estimated from a set of images acquired from a phantom filled with CuSO_4_ solution with T_1_ and T_2_ both equal to about 140 ms at the working frequency and T_R_ = 500 ms, which is an experimental condition comparable to the 8.9 mT measurements. The spatial resolution was 2x2x5.5 mm^3^ for 65x11 phase encoding steps and N_FE_ = 65, with frequency encoding gradient strength of 73 Hz/cm, NEX = 1, and RF coil bore of 10 cm. The authors report SNR of 25. To compare the two systems, the formulas (1) and (2) are used to scale the SNR measured by the 8.9 mT system to the 2 mT one. The normalization factors for the different RF coil sizes, image resolutions, FOV and BW will be considered, starting from images with different echo times (T_E_ in the present work is shorter) but the same repetition time, so that the total acquisition time will be the same. To properly scale the results obtained with the 3 mm isotropic resolution, the VLF bandwidth is increased by a factor of 1.3 (the ratio of the two FOVs) assuming to maintain 260 Hz/cm reading gradient intensity (only 73 Hz/cm are used in the 2 mT system). The sequence dependent factor (1) is 0.85 while the geometric factor for the RF coil (2) is given by the ratio of the Rx coils radii (10/8)^−3/2^ = 0.72. Thus the expected NEX = 1 performance for the 8.9 mT VLF-MRI, scaled to be comparable with the geometry and acquisition parameters of [17], is SNR = 43.

It is worthwhile to examine whether this result can be explained by the different magnetic field strengths used in the two experiments. Since in both cases the coil noise dominates sample and environmental contributions, the expected SNR behavior is $SNR\propto B_{0}^{3/4}B_{p}$ with prepolarization and $SNR\propto B_{0}^{7/4}$ without [14]. Compared to a system with *B*_0_ = 2 mT, the *B*_*p*_ = 100 mT pulses should provide a SNR increase by a factor 100/2 = 50, while changing the background static field to *B*_0_ = 8.9 mT should provide a SNR increase by a factor (8.9/2)^7/4^ = 14. When properly compared, the 8.9 mT system is expected to have a 14/50 = 0.3 times smaller SNR while the observed ratio is 43/25 ∼ 1.7, six times larger. The T_E_ difference between the two experiments can not justify such big difference. Conversely, the more efficient use of the sequence time, due to the absence of dead times related to the pre-polarization coil switching should have an important role in explaining the observed results.

### Comparison with the ULF-MRI system in [10]

The Los Alamos system [10] is chosen as an example of existing ULF MEG-MRI device to be compared with the 8.9 mT system. Specifically, the experiment in [10] is selected since the authors report a higher resolution than the Aalto device [6], with a comparable image quality and acquisition time. To scale the 8.9 mT results, the receiver coil factor (2) is calculated enlarging the RF coil (with an inner diameter of 8 cm) by a factor of 3 to contain a human head. This gives a SNR reduction by a factor of 5. Starting from the 3x3x3 mm^3^ VLF acquisitions, Δz has to be increased by a factor of two (from 3 to 6 mm), N_PE1_xN_PE2_xN_FE_ should change from 32x32x32 to 51x9x90 while the BW has to be increased by a factor of 2.1 (to get a 20 cm FOV). The factor (1) gives an SNR increase by a factor of 1.55 and, together with the geometrical factor (2), an overall SNR change by a factor of 1.55/5 ∼ 0.31 is expected. This SNR reduction could be partially compensated by the addiction of a second receiver coil in quadrature configuration with a consequent SNR increase by a factor of $\sqrt{2}$. This solution isn’t adopted in the test system because of its small size even thought it would be easy to implement in a human-sized device. In total, a SNR reduction factor of 0.42 should be expected relative to the small-scale demonstration.

Starting from the SNR measured for the ex-vivo rabbit brain with T_R_ = 500 ms, a SNR = 16 for a full brain acquisition with NEX = 1 and a total acquisition time T_acq_ = N_PE1_xN_PE2_xT_R_ = 3.8 min is expected. When compared with the SNR = 10 and T_acq_ = 10 min reported in [10], the 8.9 mT system seems to provide a similar SNR with a considerably shorter acquisition time. Specifically, the 8.9 mT system is expected to provide images with SNR = 22, above the criterion SNR > 20 for adequate anatomical imaging [11], with NEX = 2 and a T_acq_ = 7.6 min. The above conclusion is drawn on the unfixed post-mortem rabbit data with T_R_ = 500 ms and depends on the specific values of relaxation times in the sample, which set the T_R_ and the total acquisition time. However, when comparing the T_R_ = 500 ms images of Fig 10 with the T_R_ = 300 ms ones of Fig 11, more details can be appreciated in the latter, due to T_1_ contrast effects. This suggests that T_1_ of the ex-vivo rabbit brain should be smaller than the shorter of the T_R_ values used, i.e. similar to the values of in-vivo human white and grey matter at 300 kHz which are 140 and 220 ms respectively [35].

### Strategies to improve the system performance

The results of the above comparisons suggest that a VLF isotropic 3 mm resolution MRI scan of the human brain, with SNR adequate for anatomical imaging and manageable total scan time, could be achievable without necessarily increasing the static background B_0_ field.

Higher values of SNR or better spatial resolution could be obtained without increasing the B_0_ value by: (i) averaging the signal, (ii) reducing the BW (with a more accurate main coil design or compensating geometrical distortion in the image through a software calibration like in [17]), (iii) improving the Rx coil performance increasing the Q factor, (iv) improving B_0_ stability. The latter could be achieved using a more stable (and considerably more expensive) current generator. Alternatively, the effect of B_0_ fluctuations could be corrected by measuring B_0_ and compensating the observed frequency changes either in real time (frequency shift of the transceiver reference frequency) or a posteriori during image reconstruction. The correction procedure can be based on the use and excitation of a separate sample with a separate RF excitation coil. Recently a very sophisticated version of this strategy, correcting for inhomogeneities both in space and time, was considered in HF-MRI [36].

VLF-MRI can use conventional sequences of HF-MRI to achieve performance improvements over spin echo (e.g., gradient echo, multi echo, driven equilibrium, steady state, etc.) like recently demonstrated at 6.5 mT in [37]. It would be hard to quantify the expected SNR improvement, as it would depend on the contrast strategy of interest. Thus, this quantification will not be discussed in this paper but it would be an interesting issue to be addressed by future experiments.

Possible SNR increases determined by higher B_0_ values, provided that compatibility with the MEG environment is still granted, are now discussed. In this experiment, *B*_0_ is limited to 8.9 mT to allow the RF signal to enter the superinsulation layer of the liquid helium cryostat. This field value allowed to test both cryogenic [20] and non-cryogenic acquisition schemes. Based on the experience of other research groups it is possible to assume that *B*_0_ = 20 mT [10, 16, 25] or even higher [38] should still be compatible with a SQUID-based MEG system. A 20 mT magnetic field, if generated by a closed coil configuration, would still allow a reasonable power dissipation and magnet size in a system for imaging the human head. A field upgrade by a factor *f* = 2 would roughly increase the SNR by *f* ^7/4^ = 3.4, making the 2 mm isotropic resolution feasible.

Finally, the possibility of reliably co-registering the VLF and HF-MRI images suggests new strategies to be used in a VLF system. As shown for the rabbit head in Fig 10, it is possible to co-register both images, although only a part of the rabbit head is recorded by the VLF system. Accordingly, it would be possible to reduce the voxel size below 3 mm without dramatically increasing the total acquisition time by recording a few VLF-2D acquisitions (with slice-selection pulses) at higher resolution. These images might provide enough information to be co-registered with whole brain HF-MRI scans, e.g. using algorithms based on Mutual Information which were successfully adopted for the co-registration of partial brain volumes over complete ones [39]. In the perspective of an integrated MEG-MRI device, fast 2D VLF-MRI scans might improve the co-registration between the functional (from MEG) and anatomical (from HF-MRI) images, increasing the spatial resolution of MEG.

## Conclusion

In this work a VLF-MRI system, compatible with the MEG environment and without pre-polarization, is proposed as a viable alternative to ULF-MRI devices with pre-polarization. Even static B_0_ fields as low as ∼9 mT provide performance comparable to ULF with large polarization fields. Notably, the set-up is considerably simpler than ULF-MRI and would still allow imaging of patient categories that could not undergo HF scans. Although a small-scale prototype is shown here, it is designed to be scalable up to a human brain sized scanner. The study of MEG-compliant VLF systems wasn’t pursued until now even though it has some advantages. First, for *B*_0_ ∼ 9*mT*, the VLF scanner provides acceptable SNR with reduced acquisition times as compared to state-of-the art ULF systems. Second, image reconstruction is straightforward since all 2D and 3D sequences from HF-MRI can be used, without dealing with concomitant gradient effects. Finally, increasing the static field strength, within the range of MEG compatibility, would directly improve the image quality and/or resolution and shorten the acquisition time. It might be argued that, at VLF, the T_1_ separation of different tissues could be reduced if compared to ULF [38]. However, it must be considered that T_1_ image contrast is a complex quantity that depends not only on the T_1_ difference between tissues but also on the spin density, the absolute value of the relaxation times, and the pulse sequence details. In this respect, the advantage of ULF over VLF MRI isn’t clearly established yet.

Could VLF-MRI be a viable alternative to HF-MRI to estimate the volume conductor model used for MEG localization and to locate sources into the brain? The answer is probably not, at least for *B*_0_ below 10 mT. The quality and resolution of images that can be acquired in a reasonable scan time is too low to reach this goal. The VLF images can however be very useful navigator images to accompany high field MRI scans, reducing localization uncertainties of the MEG approach. The use of higher values for the static B_0_ field within the range of MEG compatibility holds the potential for eliminating high field MRI reference scans in the future.
